# Different clinical characteristics and outcomes of adult hospitalized SARS-CoV-2 pneumonia patients complicated by cardiovascular events during the first, delta and omicron waves of COVID-19

**DOI:** 10.3389/fepid.2024.1342917

**Published:** 2024-04-18

**Authors:** Lynn P. Roser, Harideep Samanapally, T’shura Ali, Qian Xu, Yuchen Han, Vidyulata Salunkhe, Fnu Deepti, Trevor McGuffin, Emma C. Huang, Stephen Furmanek, Alex Glynn, Julio Ramirez, Christopher M. Jones, Ramesh Mariyappa, Ryan J. Hogue, Alexander M. Williams, Justin J. Huang, Forest W. Arnold, Sean P. Clifford, Siddharth Pahwa, Maiying Kong, Jiapeng Huang

**Affiliations:** ^1^ School of Nursing, University of Louisville, Louisville, KY, United States; ^2^ Division of Infectious Diseases, Centre of Excellence for Research in Infectious Diseases (CERID), University of Louisville, Louisville, KY, United States; ^3^ Department of Bioinformatics and Biostatistics, School of Public Health and Information Sciences, University of Louisville, Louisville, KY, United States; ^4^ Department of Anesthesiology, Duke University, Durham, NC, United States; ^5^ Kornhauser Health Sciences Library, University of Louisville, Louisville, KY, United States; ^6^ Division of Transplantation, Department of Surgery, University of Louisville, Louisville, KY, United States; ^7^ Department of Anesthesiology & Perioperative Medicine, University of Louisville, Louisville, KY, United States; ^8^ Department of Cardiovascular & Thoracic Surgery, University of Louisville, Louisville, KY, United States; ^9^ Department of Pharmacology & Toxicology, University of Louisville, Louisville, KY, United States; ^10^ Center for Integrative Environmental Health Sciences, University of Louisville, Louisville, KY, United States

**Keywords:** SARS-CoV-2, COVID-19, omicron, delta, cardiac, vascular, outcome

## Abstract

**Background:**

The effects of SARS-CoV-2 have varied between significant waves of hospitalization.

**Research question:**

Are cardiovascular complications different among the first, delta and omicron waves of hospitalized COVID-19 pneumonia patients?

**Study design and methods:**

This was a multi-centre retrospective study of patients hospitalized with SARS-CoV-2 pneumonia: 632 were hospitalized during the *first wave* (March–July 2020), 1013 during the *delta wave* (September 2020–March 2021), and 323 during the *omicron wave* (January 2022–July 2022). Patients were stratified by wave and occurrence of cardiovascular events.

**Results:**

Among all hospitalized patients with cardiovascular events, patients in the omicron wave were younger (62.4 ± 14 years) than patients in the first wave (67.4 ± 7.8 years) and the delta wave (66.9 ± 12.6 years) and had a higher proportion of non-Hispanic White people than in the first wave (78.6% vs. 61.7%). For COVID-19 patients who suffered from cardiovascular events, the omicron wave patients had significantly higher neutrophil/lymphocyte ratio, white blood cell and platelet counts when compared to the first wave. Omicron wave patients had significantly lower albumin and B-type natriuretic peptide levels (only 5.8% of the first wave and 14.6% of the delta wave) when compared to either the first wave or delta wave patients. In COVID-19 patients who suffered cardiovascular events during hospitalization, mortality rate in the omicron wave (26.8%) was significantly lower than the first wave (48.3%), time to mortality for non-survivors of COVID-19 patients who suffered cardiovascular events was significantly longer in the omicron wave (median 16 days) than in the first wave (median 10 days).

**Conclusions:**

Younger and white patients were affected with cardiovascular complications more often by the omicron variant. Despite higher neutrophil/lymphocyte ratio and WBC counts, the omicron patients with cardiovascular events showed lower heart injuries, lower mortality and longer time to mortality for non-survivors when compared to the first and delta waves.

## Introduction

The worldwide COVID-19 pandemic has challenged public health infrastructure due to significant morbidity and mortality and created significant social and economic disturbances across the globe (1). Over 6.6 million deaths have been attributed to COVID-19 worldwide (2). Historically, pandemics caused by viral illness have occurred in waves, described as significant incidence spikes subsequent to the initial outbreak. Most countries experienced at least three waves of the COVID-19 pandemic, the first wave in spring 2020, the second wave in late summer and autumn 2020 and the third wave starting from November 2021 (3, 4). Viral effects differed in demographics as well as severity of illness. The second wave of COVID-19 was attributable to the highly contagious delta variant (5). Starting December 2021, the omicron variant has become the dominant variant in many countries (6). Omicron is a new variant of the severe acute respiratory syndrome coronavirus 2 (SARS-CoV2) with numerous mutations in the N-terminal domain of the viral spike protein and the receptor binding domain. This facilitated the angiotensin-converting enzyme 2 (ACE2) receptor-based cell entry. Therefore, when compared to the delta variant, the omicron variant is 2–3 times more transmissible, which explained peak number of daily new cases of COVID-19 and the quick spread in most of western countries (7).

The effects of COVID-19 on the health and well-being of patients reach far beyond the pulmonary system. As has been reported in numerous studies, COVID-19 may cause cardiovascular complications, such as myocarditis, endothelial dysfunction, thrombotic events, acute myocardial infarction, cardiac fibrosis, arrhythmias, and dysautonomia (8–11). SARS-CoV-2 affects the cardiovascular system through infection of the myocardium, vascular tissues, and circulating cells via the host's ACE-2 receptors for the viral spike protein (10). The pandemic ongoing and the potential for increased cases in future waves have substantial implications for the cardiovascular health globally (9). A recent meta-analysis of 21 studies, including over 77,000 hospitalized COVID-19 cases, evaluated in-hospital cardiovascular events and their impact on mortality. Over 12% of hospitalized patients had cardiovascular comorbidities or risk factors, and over 14% had a cardiovascular event while hospitalized. Cardiovascular comorbidities or risk factors and the development of in-hospital cardiovascular events were significantly associated with mortality (12, 13). These results demonstrated the importance of characterizing the cardiovascular risk profile of COVID-19 patients to identify higher risk patients for effective clinical management. However, there are scarce data on omicron's cardiac involvement especially its comparisons with the first and delta waves.

The University of Louisville COVID-19 Cardiovascular Research Group of the Center of Excellence for Research in Infectious Diseases (CERID) studied the characteristics and outcomes of COVID-19 patients who suffered cardiovascular events and identified risk factors in white and African American populations during the first wave of COVID-19 in Louisville, KY, USA (14). We also evaluated the electrocardiographic and echocardiographic features and their associations with clinical outcomes in this population during the first wave (15, 16). The objective of the present study is to compare the clinical characteristics and outcomes of patients hospitalized with SARS-CoV-2 community-acquired pneumonia (CAP) who suffered in-hospital cardiovascular events in the first wave (March 2020–July 2020), delta wave (September 2020–March 2021) and omicron wave (January 2022–July 2022).

## Methods

Informed consent was not required per the exemption by the Institutional Review Board (IRB).

### Study design, subjects, and setting

This investigation is a multi-center retrospective observational cohort study of patients hospitalized with SARS-CoV-2 CAP at eight adult, acute-care hospitals in Louisville, KY, USA. COVID-19 hospitalizations between March 2020 and July 2022 were included in this analysis. Patients with (1) a positive reverse transcription-polymerase chain reaction (RT-PCR) for SARS-CoV-2, (2) symptoms including fever, cough, or shortness of breath, and (3) an infiltrate on chest imaging were defined as having SARS-CoV-2 CAP. Patients were followed until hospital discharge or in-hospital death. For this analysis, a cardiovascular event during hospital was defined as any of the following conditions: development of cardiogenic shock, heart failure, acute myocardial infarction, cardiomyopathy, myocarditis, a new, serious arrhythmia, acute worsening of long-term arrhythmia, cerebrovascular accident, pulmonary embolism, deep vein thrombosis, pulmonary edema, or cardiac arrest occurring after hospital admission confirmed by an attending physician at each participating hospital. A new, serious arrhythmia, acute worsening of long-term arrhythmia was defined as any new or worsening arrhythmia that was not sinus rhythm including ventricular, junctional, and atrial arrythmia. Cardiomyopathy was defined as one of the following diagnoses: dilated cardiomyopathy, hypertrophic cardiomyopathy, restrictive cardiomyopathy, arrhythmogenic cardiomyopathy or unclassified cardiomyopathy. Acute myocardial infarction was defined as ECG evidence of ST segment elevation or depression, and/or cardiac troponin values at least one value above the 99th percentile of upper reference limit. This cohort represents real world settings where diagnosis and management decisions are made by practicing physicians. The three waves of COVID-19 were defined by the most dominant variant present at the time of the COVID-19 diagnosis in Louisville, KY, USA: the first wave (March 2020–July 2020), delta wave (September 2020–March 2021) and Omicron Wave (January 2022–July 2022).

### Human subjects protection

The study was approved by the Institutional Review Board (IRB) at the University of Louisville Human Subjects Research Protection Program Office (IRB number 20.0257) and by the research offices at each participating hospital. Informed consent was not required per the exemption by the IRB.

### Study coordinating centre

CERID, located in the University of Louisville Division of Infectious Diseases, implemented all operations of the study. Members of CERID developed the case report form and the study database, collected data from hospital electronic medical records (EMRs), entered data into database software, and performed quality control of collected data by initiating and resolving queries (17). Data were collected and managed using REDCap (Research Electronic Data Capture) tools hosted at the University of Louisville Division of Infectious Diseases.

### Data collection

Data collected from EMRs included COVID-19 test results; current medications; demographics, medical and social history; physical examination; signs and symptoms of illness; and laboratory, radiologic, and microbiologic findings. Disease management and therapies were noted as well as clinical course, in-hospital complications, and outcomes.

### Demographic variables and comorbidities

Demographic data—including age, sex, height, and weight to calculate body mass index (BMI) and race or ethnicity of each patient—were captured to fully evaluate the population sample. Patients were grouped into the following categories: Hispanic; non-Hispanic black; non-Hispanic white; non-Hispanic others. Cardiovascular comorbidities collected included a history of heart failure, congestive heart failure, peripheral vascular disease, cerebrovascular disease, coronary artery disease, essential arterial hypertension, hyperlipidemia, prior myocardial infarction, prior PTCA/CABG, atrial fibrillation, prior deep vein thrombosis and prior pulmonary embolism. Current medication use related to cardiovascular history included aspirin, beta-blockers, angiotensin converting enzyme (ACE) inhibitors, anticoagulants, antiplatelets, statins, spironolactone/eplerenone, calcium channel blockers and angiotensin receptor blockers (ARBs).

### Clinical and laboratory variables

Clinical and laboratory data were collected within 48 h of admission or during intensive care unit admission and included partial pressure of oxygen/fraction of inspired oxygen (PaO_2_/FiO_2_) ratio, oxygen saturation/fraction of inspired oxygen (SpO_2_/FiO_2_) ratio, BMI, and arterial blood gases (ABGs) when available. Other laboratory data collected included hemoglobin; hematocrit; platelets; white blood cell count (WBC); neutrophil count/percentage; lymphocyte count/percentage; neutrophil/lymphocyte ratio (NLR); serum potassium, glucose, blood urea nitrogen (18), creatinine, albumin, and bilirubin; alanine aminotransferase (ALT) and aspartate aminotransferase (AST) levels; AST/ALT ratio, international normalized ratio (INR) measurement; procalcitonin, D-dimer level; B-type natriuretic peptide (BNP) and N-terminal pro B-type natriuretic peptide (NT-proBNP); interleukin-6 (IL-6); and C-reactive protein (CRP).

Disease management and in-hospital therapies collected included the use steroids, hydroxychloroquine, azithromycin, remdesivir, monoclonal antibodies, heparin, warfarin, ACE inhibitors, angiotensin receptor blockers (ARBs), statin, metformin, plasma therapy, nasal cannula (non-high flow and high-flow), extracorporeal membrane oxygen (ECMO), prone position, neuromuscular blockade/artificial paralysis, inhaled pulmonary vasodilators, inotropes, insulin, non-invasive mechanical Ventilation (NIMV), invasive mechanical ventilation (IMV), vasopressors, antithrombotic prophylaxis and systemic steroids.

The main outcome variables were in-hospital mortality, length of stay (survivors), and days to mortality (non-survivors). Length of stay was defined as the time between admission and discharge from the hospital in days. Days to mortality was defined as the time between admission and in-hospital mortality in days.

### Statistical analyses

Descriptive statistics was used to compare demographics, comorbidities, laboratory tests, disease management and therapies and clinical outcomes across the three different COVID-19 waves and development of in-hospital cardiovascular events. Continuous variables were summarized as means and standard deviations (SD) for each group, and categorical variables were summarized as frequencies and percentages for each group. For continuous variables, post-hoc t-tests or Mann-Whitney were performed to examine differences between groups; and one-way analysis of variance (ANOVA) or Kruskal-Wallis were performed to examine differences among groups. For categorical variables, Chi-squared tests were used if the number of observations in each cell was >5; otherwise, Fisher's exact tests were used.

Pairwise and three groups comparisons were carried out for the following group combinations: (1) first wave (March 2020–June 2020) vs. delta wave (September 2020–March 2021) vs. omicron wave (January 2022–July 2022) among patients who suffered cardiovascular events; (2) first wave (March 2020–June 2020) vs. delta wave (September 2020–March 2021); (3) Delta wave (September 2020–March 2021) vs. omicron wave (January 2022–July 2022) and (4) first wave (March 2020–June 2020) vs. omicron wave (January 2022–July 2022).

Kaplan–Meier estimators with log-rank tests or weighted Kaplan-Meier tests were conducted to compare survival times among patients with cardiovascular events for three waves (19). Statistical analyses were conducted using R version 4.0.2 (R Foundation for Statistical Computing, Vienna, Austria, 2020). *P*-values <0.05 were considered statistically significant.

## Results

During the first wave, 120 (18.99%) patients developed in-hospital cardiovascular events and 512 patients who did not; during the delta wave, 180 (17.77%) patients who developed in-hospital cardiovascular events and 833 patients who did not; and lastly, during the omicron wave, 56 (17.33%) patients developed in-hospital cardiovascular events and 267 patients who did not.

Patient demographics stratified by COVID-19 waves and occurrence of cardiovascular event(s) are summarized in Table 1A. Among all hospitalized patients with cardiovascular events, patients in the omicron wave were younger (62.4 ± 14 years) than patients in the first wave (67.4 ± 7.8 years) and the delta wave (66.9 ± 12.6 years) and had a higher proportion of non-Hispanic White people than in the first wave (78.6% vs. 61.7%).

**Table 1:** Demographics, cardiovascular complications and comorbidities of three waves of COVID-19 patients who developed cardiovascular events during hospitalization. First wave (March 2020–July 2020); Delta wave (September 2020–March 2021); Omicron wave (January 2022–July 2022).

	First wave (*N* = 120)	Delta wave (*N = *180)	Omicron wave (*N* = 56)	*P* value Overall	*P* value First vs. delta	*P* value Delta vs. omicron	*P* value First vs. omicron
A: Demographics							
Age (mean ± SD)	67.4 ± 14.6	66.9 ± 12.6	62.6 ± 14	0.078	0.747	0.042*	0.048*
BMI (mean ± SD)	31.5 ± 7.9	32.4 ± 8.5	30.8 ± 10.2	0.415	0.37	0.623	0.287
Male sex (%)	68 (56.7%)	109 (60.6%)	35 (62.5%)	0.707	0.582	0.917	0.57
Non-Hispanic White (%)	74 (61.7%)	125 (69.4%)	44 (78.6%)	0.072	0.203	0.249	0.04*
Non-Hispanic African American (%)	41 (34.2%)	48 (26.7%)	11 (19.6%)	0.113	0.206	0.377	0.074
Non-Hispanic other (%)	5 (4.1%)	7 (3.9%)	1 (1.8%)	0.887	0.815	>0.999	>0.999
B: In-hospital cardiovascular complications							
Heart failure	21 (17.5%)	31 (17.2%)	11 (19.6%)	0.915	>0.999	0.831	0.894
Acute myocardial infarction	15 (12.5%)	29 (16.1%)	11 (19.6%)	0.446	0.484	0.681	0.31
Acute cardiogenic pulmonary edema	13 (10.8%)	15 (8.3%)	2 (3.6%)	0.281	0.598	0.374	0.149
New, serious arrhythmia	52 (43.3%)	69 (38.3%)	21 (37.5%)	0.635	0.456	>0.999	0.57
Acute worsening of long-term arrhythmia	19 (15.8%)	15 (8.3%)	5 (8.9%)	0.122	0.069	>0.999	0.314
Cerebrovascular accident	7 (5.8%)	12 (6.7%)	1 (1.8%)	0.425	0.961	0.311	0.439
Pulmonary embolism	6 (5%)	23 (12.8%)	11 (19.6%)	0.011*	0.042*	0.289	0.005*
Myocarditis	2 (1.7%)	1 (0.6%)	0 (0%)	NA	0.566	NA	NA
Deep vein thrombosis	6 (5%)	15 (8.3%)	6 (10.7%)	0.355	0.38	0.781	0.28
Cardiogenic shock	14 (11.7%)	6 (3.3%)	3 (5.4%)	0.018*	0.009*	0.446	0.274
Cardiac arrest	20 (16.7%)	33 (18.3%)	8 (14.3%)	0.771	0.829	0.62	0.856
Ischemic cardiomyopathy	8 (6.7%)	10 (5.6%)	2 (3.6%)	0.335	0.608	0.445	0.218
C: Comorbidities							
	First wave (*N* = 120)		Delta wave (*N* = 180)		Omicron wave (*N* = 56)		*P* value
Cardiovascular-related comorbidities							
Heart failure	37 (30.8%)		46 (25.6%)		11 (19.6%)		0.273
Congestive heart failure	32 (26.7%)		42 (23.3%)		9 (16.1%)		0.778
Peripheral vascular disease	7 (5.8%)		6 (3.3%)		2 (3.6%)		0.39
Cerebrovascular disease	24 (20%)		22 (12.2%)		7 (12.5%)		0.154
Hemiplegia	6 (5%)		2 (1.1%)		0 (0%)		NA
Coronary artery disease	35 (29.2%)		54 (30%)		12 (21.4%)		0.449
Essential arterial hypertension	83 (69.2%)		141 (78.3%)		29 (51.8%)		0.257
Hyperlipidemia	55 (45.8%)		98 (54.4%)		21 (37.5%)		0.061
Myocardial infarction	22 (18.3%)		28 (15.6%)		5 (8.9%)		0.268
Prior PTCA/CABG	17 (14.2%)		28 (15.6%)		6 (10.7%)		0.664
Atrial fibrillation	30 (25%)		33 (18.3%)		12 (21.4%)		0.381
Prior deep vein thrombosis	8 (6.7%)		16 (8.9%)		1 (1.8%)		0.171
Prior pulmonary embolism	2 (1.7%)		2 (1.1%)		0 (0%)		NA
Other comorbidities							
COVID-19 vaccination	0 (0%)		13 (7.2%)		8 (14.3%)		NA
Pulmonary comorbidity	16 (13.3%)		19 (10.6%)		6 (10.7%)		0.746
Liver disease	2 (1.7%)		7 (3.9%)		2 (3.6%)		0.588
Renal disease	40 (33.3%)		43 (23.9%)		7 (12.5%)		0.06
Diabetes	59 (49.2%)		84 (46.7%)		20 (35.7%)		0.235
Neoplastic/immunocompromised diseases	19 (15.8%)		31 (17.2%)		9 (16.1%)		0.945
Medications							
Aspirin	53 (44.2%)		64 (35.6%)		20 (35.7%)		0.291
Beta-blockers	51 (42.5%)		85 (47.2%)		21 (37.5%)		0.401
Angiotensin-converting enzyme (ACE) inhibitors	26 (21.7%)		34 (18.9%)		12 (21.4%)		0.817
Anticoagulants	31 (25.8%)		35 (19.4%)		9 (16.1%)		0.251
Antiplatelet	20 (16.7%)		19 (10.6%)		5 (8.9%)		0.245
Statins	58 (48.3%)		82 (45.6%)		26 (46.4%)		0.894
Spironolactone/eplerenone	2 (1.7%)		11 (6.1%)		4 (7.1%)		0.106
Angiotensin receptor blockers (ARBs)	15 (12.5%)		25 (13.9%)		9 (16.1%)		0.813
Calcium channel blocker	34 (28.3%)		48 (26.7%)		21 (37.5%)		0.291

PTCA, percutaneous transluminal coronary angioplasty; CABG, coronary-artery bypass grafting.

^*^and/or bolded text = *p*-value < 0.05.

A summary of each cardiovascular complication among patients who developed at least one cardiovascular event during hospitalization is shown in Table 1B. There was a significantly higher proportion of patients who developed cardiogenic shock in the first wave compared to the delta wave (11.7% vs. 3.3%; *p* = 0.009). While a significantly higher proportion of pulmonary embolism events occurred during the omicron wave compared to the first wave (19.6% vs. 5%; *p* = 0.05).

Among patients who developed in-hospital cardiovascular events, there was no significant difference across waves for comorbidities. In the delta and omicron waves, COVID-19 vaccination data were also collected. Of note, there were 13 (7.2%) and 8 (14.3%) vaccinated patients who suffered cardiovascular event in the delta wave and omicron wave respectively (Table 1C).

Clinical and laboratory biomarkers such as NLR, WBC counts, platelet counts, neutrophil percentage, lymphocyte percentage, lymphocyte counts, albumin, CRP and BNP values differed significantly among the three waves as shown in Table 2. For COVID-19 patients who suffered from cardiovascular events, the omicron wave patients had significantly higher NLR, WBC and platelet counts when compared to the first wave. Omicron wave patients had significantly lower albumin and BNP levels (only 5.8% of the first wave and 14.6% of the delta wave) when compared to either the first wave or delta wave patients. During hospitalization, the first troponin recorded was significantly different across all three waves (0.2 vs. 1.1 vs. 0.6 ng/ml; *p* = 0.06). This was mostly driven by the difference between Wave 1 and Wave D (0.2 vs. 1.1 ng/ml; *p* = 0.008). Peak troponin was also found to be significantly higher in Wave D compared to Wave 1 (2.3 vs. 0.6 ng/ml; *p* = 0.017).

**Table 2:** Laboratory results of three waves of COVID-19 patients who developed cardiovascular events during hospitalization. First wave (March 2020–July 2020); Delta wave (September 2020–March 2021); Omicron wave (January 2022–July 2022).

	First wave (*N* = 120)	Delta wave (*N* = 180)	Omicron wave (*N* = 56)	*P* value Overall	*P* values First vs. delta	*P* values Delta vs. omicron	*P* values First vs. omicron
AST/ALT ratio	1.8 ± 0.8	1.8 ± 1	1.6 ± 1	0.399	0.472	0.226	0.468
Neutrophil/lymphocyte ratio	9.3 ± 10.9	17.4 ± 26.4	15.2 ± 17.6	**0** **.** 007*	**0**.001*	0.506	**0**.031*
SaO_2_/FiO_2_ ratio	3.1 ± 1.5	2.9 ± 1.5	3.2 ± 1.5	0.446	0.327	0.289	0.756
WBC (10^3^/mm^3^)	8.5 ± 5.8	10.3 ± 6.6	11.8 ± 8.7	**0**.008*	**0**.015*	0.254	**0**.014*
Hemoglobin	12.4 ± 2.4	12.1 ± 2.2	12.3 ± 2.6	0.698	0.393	0.748	0.799
Hematocrit	37.6 ± 6.7	37.2 ± 6.5	37.3 ± 7.7	0.897	0.637	0.945	0.811
Platelets	195.6 ± 91.9	226.7 ± 122.2	245.9 ± 128.4	**0**.012*	**0**.013*	0.329	**0**.011*
Neutrophil percentage	76.1 ± 13.8	81.7 ± 10	79.1 ± 13	**0**.001*	**0**.001*	0.192	0.197
Lymphocyte percentage	14.7 ± 9.9	10.3 ± 7.4	12.9 ± 10	**<0**.001*	**<0**.001*	0.098	0.302
Neutrophil (10^3^/mm^3^)	8 ± 9.8	8.5 ± 5.7	9.2 ± 6.9	0.631	0.636	0.494	0.364
Lymphocyte (10^3^/mm^3^)	1.4 ± 3	0.9 ± 0.9	1 ± 0.7	**0**.041*	**0**.048*	0.311	0.124
Serum potassium(mmol/L)	4.1 ± 0.7	4.1 ± 0.8	4.1 ± 0.7	0.701	0.461	0.868	0.456
Glucose (mg/dl)	168 ± 93.3	184.7 ± 129.9	199.9 ± 184.6	0.283	0.199	0.567	0.226
BUN (mg/dl)	33 ± 25.2	33.6 ± 24.2	33.2 ± 29.6	0.984	0.858	0.941	0.965
Creatinine (mg/dl)	1.8 ± 1.3	2.3 ± 5.4	1.5 ± 1.1	0.291	0.189	0.06	0.172
Albumin (g/dl)	3.3 ± 0.7	3.3 ± 0.7	3.1 ± 0.6	0.057	0.416	**0**.043*	**0**.011*
Bilirubin (mg/dl)	0.8 ± 0.5	1 ± 1.1	1 ± 0.7	0.115	0.026	0.857	0.078
AST (units/L)	86.2 ± 171.9	70.9 ± 74.7	84.6 ± 138.5	0.547	0.365	0.492	0.947
INR	1.4 ± 1	1.4 ± 0.7	1.3 ± 0.4	0.495	0.96	0.067	0.241
Procalcitonin (ng/ml)	2.2 ± 6.2	16.6 ± 156.1	13.3 ± 45.3	0.586	0.284	0.823	0.105
D-dimer (µg/ml)	4,884.5 ± 13,280.7	7,216 ± 22,202.7	7,440.5 ± 20,192.6	0.619	0.321	0.957	0.512
Interleukin-6 (pg/ml)	190.8 ± 266.2	176.1 ± 501.1	234.5 ± 440	0.892	0.852	0.662	0.722
CRP (mg/L)	52 ± 70.8	93.1 ± 111.4	66.7 ± 87.9	**0**.006*	**0**.001*	0.15	0.399
ABG FiO_2_	61.8 ± 33.2	68 ± 31.6	65.4 ± 30.1	0.432	0.205	0.658	0.561
BNP (pg/ml)	2,170.8 ± 5,020.1	876.7 ± 2,105.8	127.7 ± 210.2	**0**.001*	**0**.018*	**<0**.001*	**<0**.001*
First troponin (ng/ml)	0.2 ± 0.8	1.1 ± 3.8	0.6 ± 2	**0**.06*	**0**.008*	0.319	0.149
Peak troponin (ng/ml)	0.6 ± 1.3	2.3 ± 5.8	1.7 ± 3.6	0.163	**0**.017*	0.554	0.11

AST, aspartate aminotransferase; ALT, alanine transaminase; WBC, white blood cell; INR, international normalized ratio; CRP, C reactive protein; ABG, arterial blood gas; BNP, brain natriuretic peptide.

^*^and/or bolded text = *p*-value < 0.05.

Treatment options across three waves of patients who suffered cardiovascular events during hospitalization were compared in Table 3. The proportions of patients were given the following treatments: steroids, remdesivir, monoclonal antibodies, nasal cannula (non-high flow) and non-invasive mechanical ventilation were significantly different across three waves. Specifically, during the omicron wave more patients were treated with monoclonal antibodies compared to the delta and first wave. The use of steroids, remdesivir, and nasal cannula (non-high flow) increased in both the delta and omicron wave compared to the first wave. Non-invasive mechanical ventilation increased from the first wave to the delta wave and lastly, the use of extracorporeal membrane oxygen (ECMO) increased in the omicron wave compared to the delta wave.

**Table 3:** Treatment options in three waves of COVID-19 patients who developed cardiovascular events during hospitalization. First wave (March 2020–July 2020); delta wave (September 2020–March 2021); omicron wave (January 2022–July 2022).

*N* (%)	First wave (*N* = 120)	Delta wave (*N* = 180)	Omicron wave (*N* = 56)	*P* values Overall	*P* values First vs. delta	*P* values Delta vs. omicron	*P* values First vs. omicron
Steroids	44 (36.7%)	161 (89.4%)	46 (82.1%)	**<0** **.** 001*	**0**.001*	0.793	**0**.**003**
Hydroxychloroquine	59 (49.2%)	0 (0%)	0 (0%)	NA	NA	NA	NA
Azithromycin	74 (61.7%)	77 (42.8%)	27 (48.2%)	0.186	0.085	0.761	0.451
Remdesivir	11 (9.2%)	107 (59.4%)	36 (64.3%)	**0**.001*	**<0**.001*	0.846	**<0**.001*
Monoclonal antibodies	3 (2.5%)	4 (2.2%)	17 (30.4%)	**0**.001*	>0.999	**<0**.001*	**<0**.001*
LMWH	67 (55.8%)	124 (68.9%)	33 (58.9%)	0.52	0.318	0.613	0.946
Heparin	50 (41.7%)	96 (53.3%)	33 (58.9%)	0.369	0.285	0.79	0.265
Warfarin	3 (2.5%)	13 (7.2%)	2 (3.6%)	0.211	0.116	0.531	0.656
ACE inhibitors	12 (10%)	34 (18.9%)	8 (14.3%)	0.189	0.1	0.642	0.626
ARBs	8 (6.7%)	15 (8.3%)	6 (10.7%)	0.698	0.786	0.817	0.581
Statin	45 (37.5%)	81 (45%)	27 (48.2%)	0.616	0.472	0.905	0.476
Metformin	4 (3.3%)	5 (2.8%)	2 (3.6%)	0.92	>0.999	0.673	>0.999
Plasma therapy	22 (18.3%)	35 (19.4%)	4 (7.1%)	0.155	0.959	0.062	0.109
Nasal cannula (non-high flow)	20 (16.7%)	138 (76.7%)	37 (66.1%)	**<0**.001*	**<0**.001*	0.617	**<0**.001*
High-flow nasal cannula	65 (54.2%)	81 (45%)	24 (42.9%)	0.587	0.42	0.971	0.502
Extracorporeal membrane oxygen (ECMO)	1 (0.8%)	1 (0.6%)	3 (5.4%)	0.057	>0.999	**0**.047*	0.104
Prone position	27 (22.5%)	22 (12.2%)	6 (10.7%)	0.083	0.067	0.972	0.172
Neuromuscular blockade/artificial paralysis	39 (32.5%)	40 (22.2%)	15 (26.8%)	0.324	0.17	0.706	0.694
Inhaled pulmonary vasodilators	18 (15%)	37 (20.6%)	6 (10.7%)	0.284	0.386	0.223	0.658
Inotropes	31 (25.8%)	47 (26.1%)	8 (14.3%)	0.315	>0.999	0.194	0.229
Insulin	68 (56.7%)	104 (57.8%)	30 (53.6%)	0.958	0.999	0.869	0.944
Non-invasive mechanical ventilation (NIMV)	15 (12.5%)	56 (31.1%)	13 (23.2%)	**0**.012*	**0**.005*	0.49	0.193
NIMV within 24 h. of admission	8 (6.7%)	21 (11.7%)	6 (10.7%)	0.419	0.267	>0.999	0.581
Invasive mechanical ventilation (IMV)	70 (58.3%)	86 (47.8%)	19 (33.9%)	0.19	0.368	0.309	0.1
IMV within 24 h. of admission	32 (26.7%)	30 (16.7%)	9 (16.1%)	0.188	0.122	>0.999	0.292
Vasopressors	57 (47.5%)	83 (46.1%)	13 (23.2%)	0.091	0.97	0.054	0.054
Antithrombotic prophylaxis	99 (82.5%)	0 (0%)	0 (0%)	NA	NA	NA	NA
Systemic steroids	45 (37.5%)	0 (0%)	0 (0%)	NA	NA	NA	NA

LMWH, low weight molecular heparin.

^*^and/or bolded text = *p*-value < 0.05.

Clinical outcomes stratified by waves and cardiovascular events are shown in Table 4A,B. Among COVID-19 patients who developed a cardiovascular event during hospitalization, mortality rate was significantly lower during the omicron wave compared to the first wave (26.8% vs. 48.3%; *p* = 0.011). In COVID-19 patients who did not develop a cardiovascular event during hospitalization, mortality rate significantly lower during the omicron wave compared to the delta wave (5.6% vs. 13.1%; *p* = 0.001).

**Table 4:** Clinical outcomes including death, hospital stay for survivors and days to mortality for non-survivors in three waves of COVID-19 patients who developed cardiovascular events during hospitalization. First wave (March 2020–July 2020); Delta wave (September 2020–March 2021); Omicron wave (January 2022–July 2022).

	First wave (*N* = 120)	Delta wave (*N* = 180)	Omicron wave (*N* = 56)	*P* value Overall	*P* value First vs. delta	*P* value Delta vs. omicron	*P* value First vs. omicron
A: COVID-19 patients who developed a cardiovascular event during hospitalization							
Death (%)	58 (48.3%)	73 (40.6%)	15 (26.8%)	**0** **.** 025*	0.226	0.089	**0**.011*
LOS for survivors (days)^a^	10.5 (6, 18)	9 (6, 17.5)	8 (5, 15)	0.379	0.576	0.212	0.095
Days to mortality for non-survivors (days)^a^	10 (6, 15)	14 (9, 20)	16 (10, 20)	**0**.022*	**0**.008*	0.653	**0**.021*
B: COVID-19 patients who did NOT develop a cardiovascular event during hospitalization							
Death (%)	49 (9.6%)	109 (13.1%)	15 (5.6%)	**0** **.** 002*	0.063	**0**.001*	0.077
LOS for survivors (days)^a^	6 (3, 12)	7 (4, 11.3)	6 (4, 10)	0.377	0.773	0.202	0.184
Days to mortality for non-survivors (days)^a^	8 (5, 12)	13 (8, 22)	13 (7.5, 24.5)	0.115	**0**.004*	0.954	0.077

LOS, length of stay.

^a^ Median (interquartile range: *Q*_1_, *Q*_3_).

^*^and/or bolded text = *p*-value < 0.05.

There was no significant difference in the hospital length of stay for survivors of both COVID-19 patients who did and who did not develop a cardiovascular event during hospitalization across three waves (Table 4B). However, time to mortality for non-survivors of COVID-19 patients who developed an in-hospital cardiovascular event was significantly longer in the omicron wave compared to both the first wave (median 16 vs. 10 days; *p* = 0.021) and the delta wave (median 10 vs. 14 days; *p* = 0.008) (Table 4A). In addition, among patients who did not develop a cardiovascular event during hospitalization also had a significantly longer time to mortality for non-survivors during the delta wave compared to the first wave (median 8 vs. 13 days, *p* = 0.004) (Table 4B).

**Figure 1:**
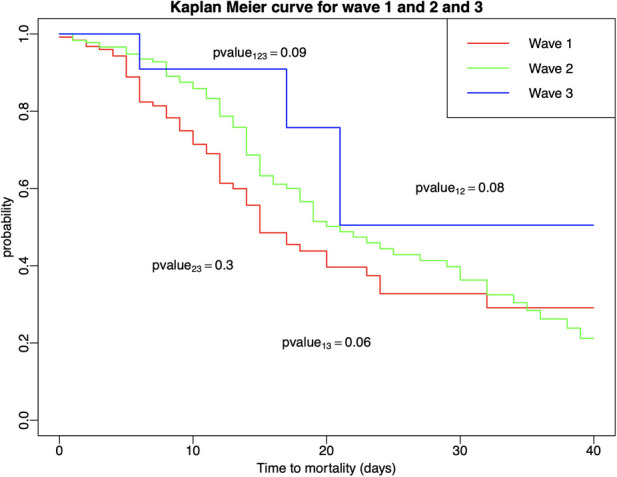
Kaplan–Meier estimators with log-rank tests were conducted to compare survival times among patients who developed in-hospital cardiovascular events across and within the three COVID-19 waves.

Kaplan–Meier curves and log-rank tests revealed that there was no significant difference in survival among three waves for COVID-19 patients who suffered cardiovascular events (Figure 1).

**Figure 2:**
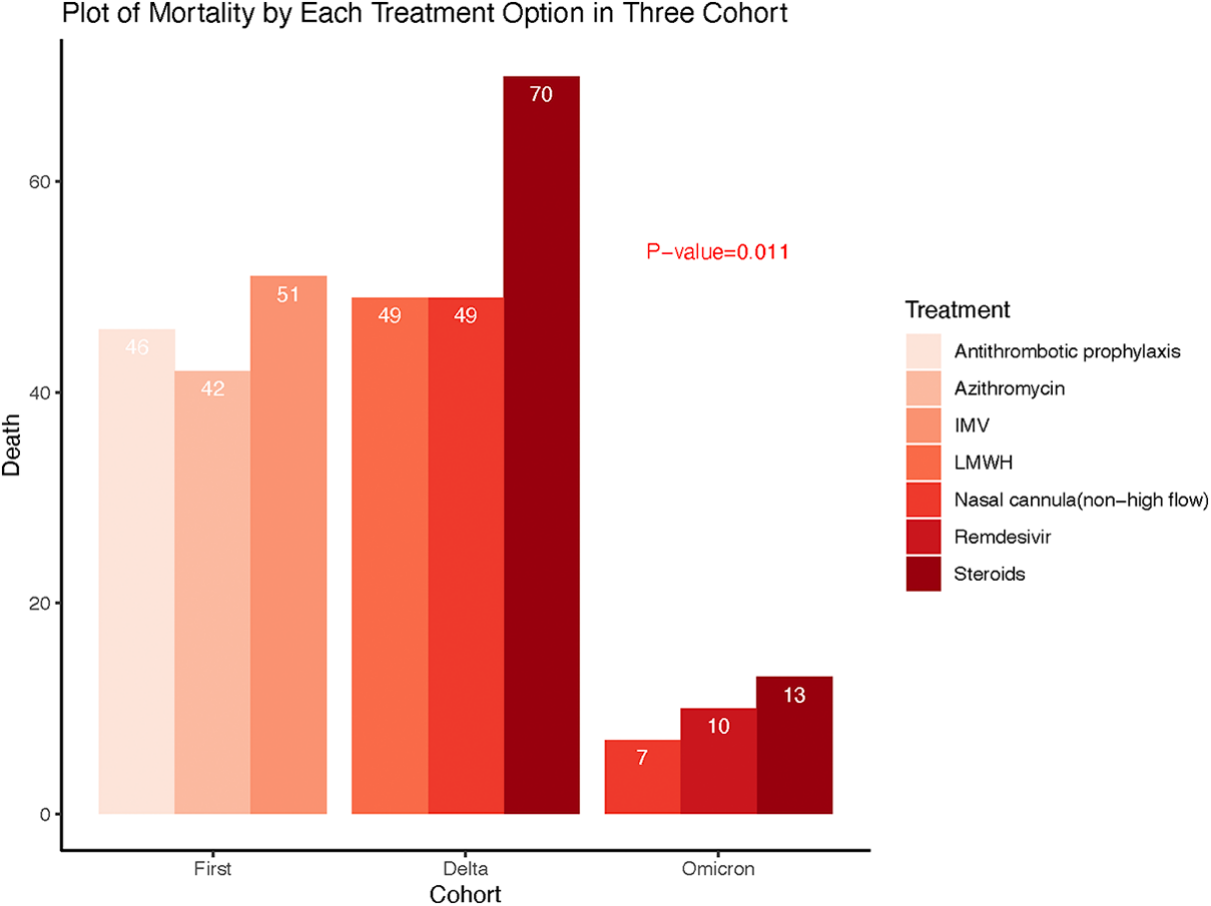
Comparison of mortality rates associated with the top3 treatments between and across three waves of COVID-19.

Table 5 furthermore demonstrated the most selected treatments as well as the corresponding mortality rates for each treatment option across the three COVID-19 waves of patients who developed cardiovascular events during hospitalization. The mortality rate of patients who received antithrombotic prophylaxis, azithromycin, and invasive mechanical ventilation (IMV) in the first wave are 46 (46.5%), 42 (56.8%) and 51 (72.9%) respectively. Steroids and nasal cannula (non-high flow) were highly used in both the delta and omicron waves. During the delta wave, 70 (43.5%) patients died who received steroids while during the omicron wave, 13 (28.3%) patients died received steroids. Forty-nine (35.5%) patients who received nasal cannula (non-high flow) died during the delta wave, however, 7 (18.9%) patients died in omicron wave. During the delta wave, 49 (39.5%) patients who received LMWH died, while, 10 (27.8%) patients who received Remdesivir died during the omicron wave. There were significant differences in mortality rates for the Top 3 treatment options among the three waves as shown in Figure 2.

**Table 5:** Mortality rates for each treatment option in three waves of COVID-19 patients who developed cardiovascular events during hospitalization. First wave (March 2020–July 2020); delta wave (September 2020–March 2021); omicron wave (January 2022–July 2022).

First wave (*N* = 120)				Delta wave (*N* = 180)				Omicron wave (*N* = 56)			
Treatment	Death (*N*)	Mortality (%)	Total got Trt	Treatment	Death (*N*)	Mortality (%)	Total got Trt	Treatment	Death (*N*)	Mortality (%)	Total got Trt
Antithrombotic prophylaxis	**46**	**46** (**46.5%)**	**99**	Steroids	**70**	**70** (**43.5%)**	**161**	Steroids	**13**	**13** (**28.3%)**	**46**
Azithromycin	**42**	**42** (**56.8%)**	**74**	Nasal cannula (non-high flow)	**49**	**49** (**35.5%)**	**138**	Nasal cannula (non-high flow)	**7**	**7** (**18.9%)**	**37**
IMV	**51**	**51** (**72.9%)**	**70**	LMWH	**49**	**49** (**39.5%)**	**124**	Remdesivir	**10**	**10** (**27.8%)**	**36**
Insulin	39	39 (57.4%)	68	Remdesivir	48	48 (44.9%)	107	LMWH	9	9 (27.3%)	33
LMWH	37	37 (55.2%)	67	Insulin	53	53 (51%)	104	Heparin	10	10 (30.3%)	33
High-flow nasal cannula	35	35 (53.8%)	65	Heparin	40	40 (41.7%)	96	Insulin	12	12 (40%)	30
Hydroxychloroquine	36	36 (61%)	59	IMV	60	60 (69.8%)	86	Azithromycin	8	8 (29.6%)	27
Vasopressors	44	44 (77.2%)	57	Vasopressors	59	59 (71.1%)	83	Statin	5	5 (18.5%)	27
Steroids	34	34 (59.6%)	57	Statin	31	31 (38.3%)	81	High-flow nasal cannula	10	10 (41.7%)	24
Heparin	29	29 (58%)	50	High-flow nasal cannula	45	45 (55.6%)	81	IMV	12	12 (63.2%)	19
Statin	17	17 (37.8%)	45	Azithromycin	34	34 (44.2%)	77	Monoclonal antibodies	4	4 (23.5%)	17
Neuromuscular blockade/artificial paralysis	28	28 (71.8%)	39	NIMV	35	35 (62.5%)	56	Neuromuscular blockade/Artificial paralysis	10	10 (66.7%)	15
IMV on hospitalization	24	24 (75%)	32	Inotropes	32	32 (68.1%)	47	NIMV	5	5 (38.5%)	13
Inotropes	24	24 (77.4%)	31	Neuromuscular blockade/Artificial paralysis	31	31 (77.5%)	40	Vasopressors	8	8 (61.5%)	13
Prone position	15	15 (55.6%)	27	Inhaled pulmonary vasodilators	12	12 (32.4%)	37	IMV on hospitalization	5	5 (55.6%)	9
Plasma therapy	11	11 (50%)	22	Plasma therapy	15	15 (42.9%)	35	ACE inhibitors	1	1 (12.5%)	8
Nasal cannula (non-high flow)	12	12 (60%)	20	ACE inhibitors	6	6 (17.6%)	34	Inotropes	6	6 (75%)	8
Inhaled pulmonary vasodilators	8	8 (44.4%)	18	IMV on hospitalization	19	19 (63.3%)	30	ARBs	0	0 (0%)	6
NIMV	9	9 (60%)	15	Prone position	18	18 (81.8%)	22	Prone position	6	6 (100%)	6
ACE inhibitors	5	5 (41.7%)	12	NIMV on hospitalization	12	12 (57.1%)	21	Inhaled pulmonary vasodilators	1	1 (16.7%)	6
Remdesivir	6	6 (54.5%)	11	ARBs	4	4 (26.7%)	15	NIMV on hospitalization	3	3 (50%)	6
ARBs	5	5 (62.5%)	8	Warfarin	5	5 (38.5%)	13	Plasma therapy	1	1 (25%)	4
NIMV on hospitalization	6	6 (75%)	8	Metformin	1	1 (20%)	5	ECMO	2	2 (66.7%)	3
Metformin	1	1 (25%)	4	Monoclonal antibodies	2	2 (50%)	4	Warfarin	0	0 (0%)	2
Monoclonal antibodies	2	2 (66.7%)	3	ECMO	1	1 (100%)	1	Metformin	0	0 (0%)	2
Warfarin	1	1 (33.3%)	3	Hydroxychloroquine	0	0 (0%)	0	Hydroxychloroquine	0	0 (0%)	0
ECMO	0	0 (0%)	1	Antithrombotic prophylaxis	0	0(0%)	0	Antithrombotic prophylaxis	0	0(0%)	0

Trt, treatment; IMV, invasive mechanical ventilation; LMWH, low molecular weight heparin; NIMV, non-invasive mechanical ventilation; ARB, angiotensin receptor blockers; ACE inhibitors, angiotensin converting enzyme inhibitors; ECMO, extracorporeal membrane oxygen.

## Discussion

The clinical characteristics, clinical markers, and outcomes of hospitalized COVID-19 patients during the first wave of COVID-19 have been previously reported by the University of Louisville COVID-Cardiovascular Research Group (14). The current study further analysed the cardiovascular complications of the delta and omicron waves in hospitalized COVID-19 pneumonia patients. We found younger and white patients were affected with cardiovascular complications more often by the omicron variant. Despite a few elevated inflammatory markers, omicron patients with cardiovascular events showed significantly lower heart failure marker levels when compared to the first and delta waves. For clinical outcomes, the omicron wave had significantly lower mortality rate and longer time to mortality for non-survivors when compared to the first wave in COVID-19 patients with cardiovascular complications.

Cardiac injury can occur in ≥20% of the hospitalized COVID-19 patients and is associated with a 3% increase in cardiac arrest, a 19% increase in incidence of heart failure, and mortality rates that range from 8% to 69%. Risk factors for cardiac complications include age >70 years, BMI ≥30 kg/m^2^, male sex, diabetes, pre-existing cardiovascular disease, and moderate to severe pneumonia at hospital presentation. Extensive myocardial inflammation is characterized by diffuse electrocardiographic (ECG) ST elevations and depressed PR intervals with global biventricular dysfunction on echocardiogram whereas regional wall motion abnormalities and localized ECG ST-segment changes are characteristics of focal ischemic damage from macro- or microvascular thrombosis. Apical Left ventricular (LV) wall dyskinesis and mid LV wall akinesis suggests SARS-CoV-2-induced stress-induced cardiomyopathy.

Omicron accounted for >99% of COVID-19 cases and has become the predominant strain in the United States in December 2021 and. The omicron SARS-CoV2 variant is highly transmissible which might mean an increase in cases, leading to more hospitalizations, cardiovascular complications, and deaths. However, the cardiovascular effects of omicron variant are less well understood and are yet to be addressed. Cardiac complications are thought to be more frequent with the alpha and delta variants than the omicron variant. A retrospective study from Italy showed omicron patients had lower incidence of pulmonary embolism than the delta wave (20). Echocardiographic analysis of 122 COVID-19 infection during the omicron surge in New York City, USA demonstrated that right ventricular (RV) abnormality remained prevalent (34%) in hospitalized omicron patients. RV abnormality was strongly and independently associated with in-hospital mortality (21). Another small study from New York, USA showed significant myocardial injury was associated with high morbidity and mortality (22). Two cases of myocarditis in acutely infected omicron patients were reported from Israel suggested the omicron variant may cause myocarditis, and malignant arrhythmia with hemodynamic instability (23). Interestingly, a recent study revealed that young, otherwise healthy adults who had infections during the omicron wave did not exhibit impairments of cardiovascular health (24).

The current study found that the incidence of cardiovascular events was similar among the first, delta and omicron waves in the Louisville, KY, USA cohort, which is representative of the USA demographics. However, omicron affected younger and white patients more often in terms of cardiovascular complications, which was different from the first and delta waves. South Africa reported patients hospitalized in omicron wave were younger, with less comorbidities and had less need for oxygen therapy, mechanical ventilation and ICU admission when compared to the first and delta waves (25). United Kingdom study of 63,002 omicron patients found that the prevalence of symptoms differs from those of the delta variant, with reduced probability of hospital admission and less involvement of the lower respiratory tract (26). Cardiac complications were not reported in these two studies. A study of omicron COVID-19 patients with myocardial injury from the USA found the omicron wave had similar clinical characteristics as prior waves (27). The current study's findings indicate the omicron variant might affect the cardiovascular system similarly as previous SARS-CoV-2 strains despite less severe respiratory system involvement.

In this study cohort, BNP levels were much lower in the omicron patients with cardiovascular events than those of the first and delta waves. This could indicate omicron variants cause less severe myocardial stretch than prior variants. Troponin levels were significantly higher in the delta wave compared to the first and omicron wave, which may reflect severe myocardial damage or injury particularly in the setting of acute coronary syndromes like myocardial infarction among delta variants. This is consistent with recent findings that troponin elevation was more common in Delta compared to Alpha, and cumulative evidence of cardiac injury (echocardiographic abnormality and/or troponin elevation) was more common in Delta compared with Alpha or Omicron (28). In addition, mortality rate in omicron patients with cardiovascular events was significantly lower than the first wave and the time to mortality for non-survivors was significantly longer in the omicron and delta waves than in the first wave. Another study found omicron patients with myocardial injuries suffered in-hospital mortality at 23.3%, which was significantly lower in than the first (59.3%) and the delta waves (28.1%) (27). A study from South Africa demonstrated that the median length of stay decreased and mortality rate also reduced among omicron patients when compared to prior waves (25).

Previous studies showed higher inflammatory markers such as procalcitonin, D-dimer, interleukin-6 and CRP resulting in a “cytokine storm” or hyperinflammatory state, which is associated with COVID-19 disease severity (29). Hence, differences in inflammatory and other hematological biomarkers may indicate a difference in COVID-19 severity across infection waves. A single-centre study in Spain found that the majority of blood biomarkers were similar across the first and delta waves with regard to disease severity, but significant differences were found in IL-6 and D-dimer (30). Another large study from Turkey compared CRP levels of hospitalized COVID-19 patients with severe or critical disease in the first and second waves, finding significantly higher CRP levels in the second wave (31). Literature shows that cardiac inflammation and microvascular procoagulant changes were lower in the delta wave than the first wave (32). In omicron myocardial injury cases, WBC, CRP, LDH and ferritin were found to be significantly lower than the first and delta waves (27). The current study found that omicron COVID-19 patients with cardiovascular events had higher neutrophil/lymphocyte ratio and WBC counts than the first wave and there was no difference between delta wave and omicron waves in terms of inflammatory markers. There could be multiple reasons for the similarity of inflammatory markers among the three waves in this study. First, steroids are currently the standard of care and could contribute to increase in WBC and reduce the increase of inflammatory markers. Second, the differences may be due to the variations in immuno-pathogenesis among different SARS-COV-2 variants. Third, vaccination might have different efficacy/actions again different variants (26).

Hospital length of stay for survivors was similar across the three COVID-19 waves for patients with and without cardiovascular events. However, days to mortality for non-survivors with cardiovascular events were significantly longer in the delta and omicron waves when compared to the first wave. It can be hypothesized that vaccine administration, earlier administration of evolving therapeutic interventions, and increased knowledge regarding disease progression led to prognostic improvement in the outcomes of the delta and omicron wave cohorts.

Treatments for both COVID-19 and cardiovascular complications could significantly affect the clinical outcomes in different waves of COVID-19 (33–36). During the omicron wave, more patients were treated with monoclonal antibodies compared to the delta and first wave. The use of steroids, remdesivir, and nasal cannula (non-high flow) increased in both the delta and omicron wave compared to the first wave. Non-invasive mechanical ventilation increased from the first wave to the delta wave and lastly, the use of extracorporeal membrane oxygen (ECMO) increased in the omicron wave compared to the delta wave. We then explored the top 3 treatments for COVID-19 patients who suffered cardiovascular events and demonstrated there were distinct differences among the three waves. During the alpha wave, the top 3 treatments were antithrombotic prophylaxis, azithromycin and invasive mechanical ventilations; the top 3 treatments in delta wave were steroids, nasal cannula oxygen and low molecular weight heparin; the top 3 treatments in omicron were steroids, nasal cannula oxygen, and remdesivir. These differences reflected the progressive learning and changes in practice for managing this deadly pandemic by physicians, which could serve as future strategies in newer variants or viruses (37–41).

Long-term sequelae of SARS-CoV-2 infection occur in 18% to 53% of patients, including chest pain, dyspnea, palpitations/tachycardia, and postural orthostatic tachycardia. SARS-CoV-2 hypercoagulability, myocardial inflammation and injury, and microvascular thrombosis, will have significant impact on quality of life, long-term patient functional status, and mortality and require longitudinal follow-up studies and extensive research. How the omicron variants affect the long-term sequelae of COVID-19 is largely unknown and should be extensively studied because the omicron variant was responsible for the largest number of COVID-19 associated hospitalizations (11).

### Limitations

There are a few limitations to this study. The results of this retrospective study may not be generalizable to non-hospitalized patients with COVID-19, as their characteristics may differ significantly from hospitalized patients, thereby reducing external validity. Since this was a descriptive study with no regression analysis, no causal inferences can be made concerning risk factors for worse clinical outcomes in our cohort. Information bias may be present given that genomic sequencing was not used to confirm variant data for delta and omicron.

## Conclusion

The present study compared the demographic, clinical, and laboratory characteristics of COVID-19 patients with cardiovascular events in the first, delta and omicron waves of the pandemic. Younger and white patients were affected with in-hospital cardiovascular complications more often by the omicron variant. Despite higher neutrophil/lymphocyte ratio and WBC counts, the omicron patients with cardiovascular events showed lower heart injuries, lower mortality and longer time to mortality for non-survivors when compared to the first and delta waves. Prospective long-term follow-up studies are urgently needed to address long term sequelae from the omicron infections. This information can help to guide triage and treatment of at-risk groups to reduce the risk of poor clinical outcomes.

## Data Availability

The original contributions presented in the study are included in the article/Supplementary Material, further inquiries can be directed to the corresponding author.
